# Qualitative patient experiences from the Self-Blame and Perspective-Taking Intervention for eating disorders

**DOI:** 10.1186/s40337-021-00483-9

**Published:** 2021-10-14

**Authors:** Whitney Smith Hagan, Susan Mericle, Bethany J. Hunt, Jessica A. Harper, Jayme M. Palka, Sarah Pelfrey, Carrie J. McAdams

**Affiliations:** Department of Psychiatry, University of Texas Southwestern Medical Center, 5323 Harry Hines Blvd., Dallas, TX 75390-9070 USA

**Keywords:** Anorexia nervosa, Bulimia nervosa, Binge eating disorder, Perspective-taking, Recovery, Attributions

## Abstract

**Background:**

Problems in social cognition and social support contribute to eating disorders (ED). Group therapy provides an ideal format to create an experiential learning environment focused on understanding social interactions. This pilot study examined the qualitative content of the participants’ experiences in the Self-Blame and Perspective-Taking Intervention (SBPI) for ED.

**Methods:**

The SBPI was a 4-week group therapy intervention involving art therapy and psychoeducation that focused on social behaviors in ED patients. Participants received surveys immediately after the intervention and at 1 to 4 weeks after the post-intervention. Thematic analyses of qualitative feedback were performed using Braun and Clarke’s thematic analysis framework.

**Results:**

Inductive analyses revealed three main themes: (1) Developing self-acceptance through emotional reflection, (2) Changing expectations with neurosocial knowledge, and (3) Bonding and vulnerability in social interactions; all concepts intentionally targeted by the SBPI. Participants varied in their support of a guideline to exclude personal discussion of ED-related cognitions and behaviors in the group.

**Conclusions:**

As a whole, patients valued the combination of psychosocial education with group experientials focused on social behavior. Positive feedback from the SBPI suggests that adjunctive treatments that target mental-wellness constructs indirectly related to ED pathology may be helpful by allowing patients to see themselves as separable from the illness.

*Trial registration* ClinicalTrials.gov, NCT0487758. Registered 7 May 2021—Retrospectively registered. https://clinicaltrials.gov/ct2/show/NCT04877158.

## Plain English summary

Adult patients with eating disorders frequently report challenges in developing and maintaining supportive social relationships. A group therapy intervention utilizing structured art tasks in conjunction with psychoeducation about interpersonal relationships was piloted in twenty-four women with eating disorders. Participants provided written feedback about their experiences in the intervention. These responses were coded by three raters to identify consistent themes related to those experiences. Participants reported benefits related to self-acceptance and emotional regulation, valued learning about their brain and behaviors, and appreciated peer interactions in this setting. In sum, this adjunctive and structured outpatient group targeting social interactions was acceptable to outpatients with eating disorders.

## Introduction

Eating disorders (EDs) are complex mental illnesses that substantially reduce quality of life [1, 2]. Even after individuals receive treatment, reach physiological recovery, and accomplish partial or full behavioral symptom stability, EDs are associated with high rates of relapse [3, 4]. Qualitative research on the perspectives of recovered individuals have highlighted the centrality of positive relationships as both contributors to and defining features of recovery from EDs [5, 6]. Specifically, social support, meaningful connection, and building an identity outside of the ED help maintain progress towards recovery [7–10].

However, securing social support is difficult for many individuals, and may be especially challenging for individuals with EDs, as these illnesses have been associated with impairments in social function. Specifically, individuals with EDs have difficulty feeling like they belong in groups [11], show reduced eye contact in social situations [12], and have trouble building positive social relationships with a lack of social competence [13]. Patients with EDs often ruminate over interpersonal interactions and show a negative self-attribution bias [14–16]. Both individuals with anorexia nervosa (AN) and those with bulimia nervosa (BN) also demonstrate deficits in theory of mind [17]. Neural differences during social perspective-taking and social interactions are found in both AN and BN, providing further neural evidence suggesting the presence of social perceptual differences [18–21]. Interventions that alter social perceptions and behaviors may help patients to obtain social support, a factor that could improve both quality of life and psychiatric symptoms for individuals with EDs.

Many treatments for EDs include components that address social difficulties and several have focused on social cognitive targets [22]. Cognitive Remediation and Emotion Skills Training (CREST) has both individual and group formats designed to explicitly target social emotional functioning and inflexible, detail-oriented thinking styles through psychoeducation and experiential exercises [23, 24]. CREST was designed for patients with severe AN at inpatient levels of care, and its utility in outpatient settings is unknown. Radically Open Dialectical Behavioral Therapy (RO-DBT) addresses openness, social signaling, and social connectedness in AN using both individual therapy and weekly skills groups, within the conventional format for DBT [25, 26]. Finally, both group and individual versions of interpersonal psychotherapy have similar efficacy to enhanced cognitive behavioral therapy (CBT-E) transdiagnostically in EDs, suggesting treatment focusing on social interactions may be effective in these illnesses [27].

Patients with EDs often find the transition to outpatient from residential treatments difficult, due to decrease in structure, development of a sense of disconnection, and loss of group momentum fostered during residential treatment [8]. Addressing challenges in engaging social support in an outpatient setting is clinically advantageous, as risk for relapse is highest in the first year following treatment [4, 28]. Few patients with EDs in our community sustain involvement in group therapy during outpatient treatment [29]. Designing groups explicitly for outpatients with EDs may provide social connection and peer support at a crucial phase in treatment.

Group interventions for EDs in outpatient settings have been shown to be feasible and acceptable [30, 31]. We propose that challenges in navigating social environments, such as impairments in emotional appraisal and difficulty receiving social support, may increase ED symptom expression. Given this etiological hypothesis, we developed an intervention targeting these domains by requiring participants in a group to engage in complex social interactions within a supportive environment in concert with education about social behaviors.

Recently, we reported on the psychological and clinical results of this intervention, finding improvements in both assessments of self-concept as well as clinical symptoms of anxiety, depression, and eating disorders [32]. Specifically, participants reported a more positive self-attribution bias, as well as increased trait and state self-esteem at the first post-intervention follow-up. In addition, symptoms of depression, anxiety, and eating disorder behaviors were significantly decreased at both the 1 to 4 weeks post-intervention and 3 to 5 months post-intervention time points.

Here, we examined qualitative feedback collected from participants after completing the SBPI intervention to better understand how participants perceived and experienced this brief group therapy. The qualitative and quantitative data were examined separately to allow for a more thorough investigation of both the feasibility and acceptability of the intervention as well as its impact on clinical and psychosocial outcomes. In addition, with the qualitative data, we recognized that participants might report thematic elements unrelated to the specific targets of the SBPI as designed by the investigators. Thus, we employed a qualitative thematic approach to inductively evaluate written feedback from the Self-Blame and Perspective-Tasking Intervention (SBPI).

## Method

### Enrollment and measures

Participants were recruited for the SBPI from existing research studies as well as local treatment programs. Participants were eligible for the study if they met current DSM-5 criteria for AN (including atypical anorexia), BN, or binge eating disorder and were appropriate for partial hospital, intensive outpatient, or outpatient care. Qualifying participants provided informed consent consistent with procedures approved by the UT Southwestern Institutional Review Board. Participants were screened with the Mini-International Neuropsychiatric Interview (MINI) and the Eating Disorder Assessment-5 (EDA-5) to confirm an eating disorder diagnosis [33, 34], intelligence was assessed with the Wechsler Abbreviated Scale of Intelligence [35], and demographic information collected. Pre and post-intervention assessments included both clinician-rated and self-report measures that addressed depression, anxiety, eating, self-esteem, and interpersonal attributions (see [36]). Qualitative questionnaires about the participants’ experiences with the SBPI were completed at the end of the last session and at the first follow-up post assessment.

### Overview of Self-Blame and Perspective-Taking Intervention (SBPI)

**Fig. 1:**
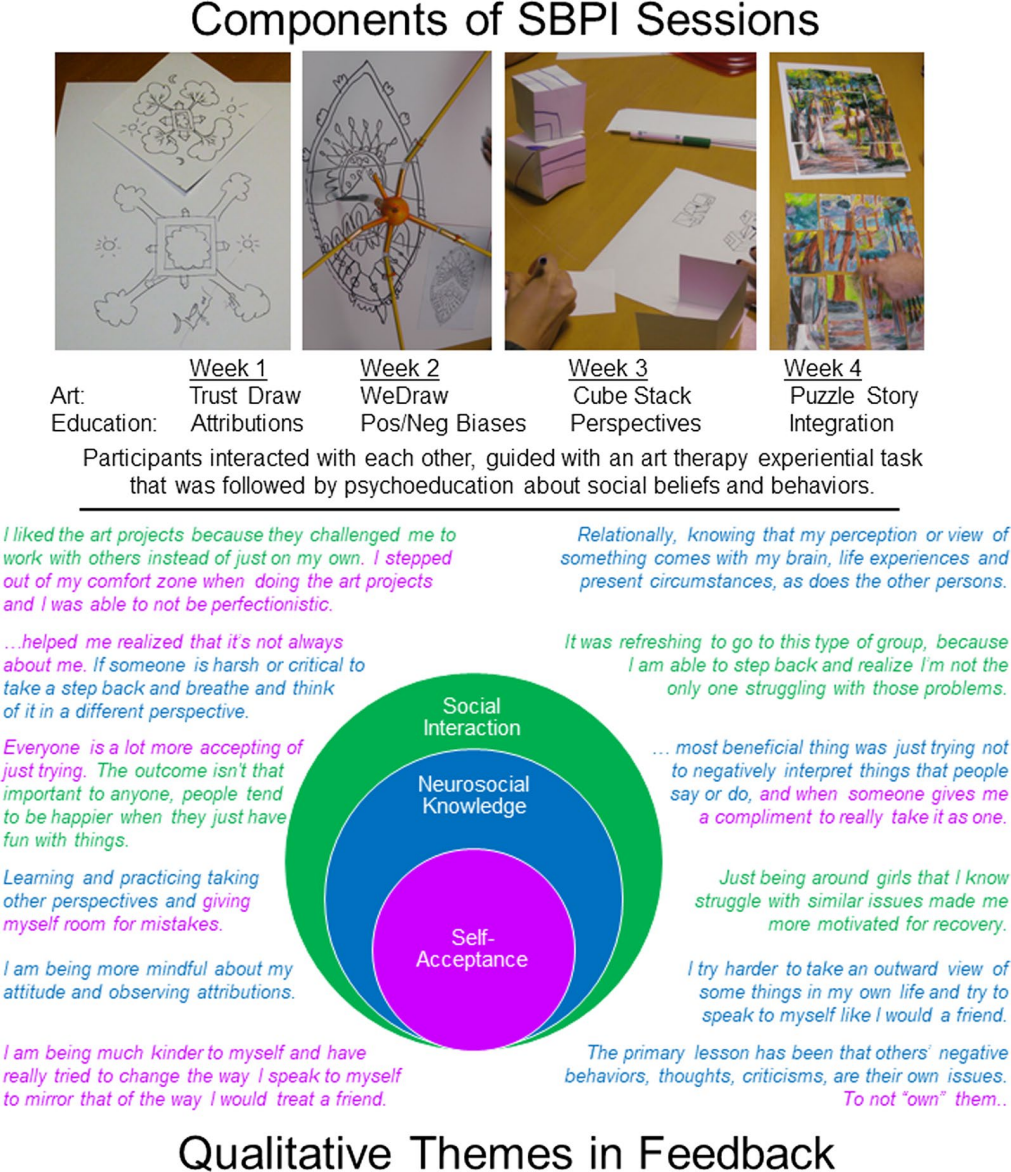
Upper panel, an overview of the art tasks and psychoeducation concepts included in each session. Lower panel, some of the qualitative feedback about the SBPI. Selected comments are color-coded according to the three themes shown in the center; comments with more than one color included codes related to different themes. The specific colored sentence fragments are selected just for illustration; comments were coded as a whole. The three themes are presented as overlapping circles, to symbolize how the emotional experience of an individual (Developing *self-acceptance* through emotional reflection) are processed using intellectual reasoning (Changing expectations with *neurosocial knowledge*), and placed in the context of real-world experiences (Bonding and vulnerability in *social interactions*)

The SBPI consisted of four 2-h weekly in-person sessions held in a group meeting room. Each session included an experiential art task, a psychoeducation group, and a homework assignment. Each of the art tasks required the group to cooperate and communicate clearly; the focus of the art tasks coordinated with psychoeducation about social function in EDs, with the specific topics selected being self-blame, positive and negative biases, and perspective-taking (upper panel, Fig. 1, additional details [32]). Four SBPI groups were offered between January 2018 and March 2019, with five to eight individuals enrolled per group. An experienced art therapist and a research assistant were present for all sessions; psychiatry residents and psychology graduate students assisted with psychoeducation using a manual developed prior to the intervention. The SBPI addressed social targets exclusively, and participants were directed to avoid discussion of ED clinical symptoms, including cognitions and behaviors. Investigators were concerned that discussion on ED cognitions and behaviors would risk creating a competitive environment that would detract from the relational goals of the intervention, as well as confound interpretation of any quantitative changes related to self-concept and psychopathology. Our decision to include this guideline in the group is investigated in this qualitative analysis.

The psychoeducation component of the SBPI was closely coordinated with the art experientials, providing both verbal and visual examples of the psychoeducation targets: interpersonal attribution biases, perspective-taking and the emotional impact on oneself in setting positive or negative expectations about interpersonal interactions. For example, self-blame was subverted by the art experientials, with the tasks designed to prevent perfectionism and result in imperfect products. As all group members shared credit for the imperfect finished product, the tendency to blame oneself for the imperfections was often challenged as other group members felt similarly, helping to create peer support bonds. Perspective-taking from alternative viewpoints was concretely demonstrated in both the art experientials including directing others with different viewpoints as well as in role-plays that required shifting one’s social perspectives. Changing background stories about hypothetical social interactions were used to demonstrate how positive and negative expectations can alter a person’s emotional reactions to an event. Finally by maintaining highly structured sessions, the group leaders were able to ensure a positive shared group experience for most participants.

### Qualitative feedback data

To gain a more nuanced understanding of the participants’ experiences, qualitative feedback about the intervention was obtained at two time points. Immediately after the final session, a short ratings questionnaire about each week’s activities included two open-ended queries: “What was the most helpful part of the intervention?” and a space for “Other comments.” At a follow-up assessment occurring 1 to 4 weeks after the SBPI, a longer open-ended questionnaire was completed. The prompts included (1) “Since finishing the intervention, what has been the primary lesson or take-away that has stuck with you?” (2) “What component of the intervention (art project, psychoeducation, activities, videos, or homework) do you think has been the most valuable? Least valuable? Why?” (3) “Are there any specific strategies that you learned that have helped you in everyday life?” (4) “What specific changes would you recommend in order to improve the intervention?” (5) “How has this intervention compared to other forms of treatment that that you have received?” (6) “Have you noticed any changes in your general attitudes or attributions since starting the intervention?” (7) “Do you think it was helpful or unhelpful that the intervention did not involve discussion of eating behaviors? Why?” and (8) “Other comments.”

### Qualitative data analysis

In order to understand the participants’ experience and views of the intervention, as well as the degree to which these experiences and views aligned with intervention targets, a four-step deductive thematic analysis was undergone following Braun and Clarke’s thematic analysis framework [37].

In the first step, prior to generating initial codes, three authors (BH, JP, & SP) reviewed the evaluation forms submitted by participants to gauge the overall impression of the intervention. This process allowed us to identify preliminary ideas about the participant feedback and how their responses could best be represented through coding. During this time, the three coders also discussed initial thoughts about codes and how best to represent participant feedback through a social cognitive lens, while also ensuring that personal biases were not introduced into the coding procedure. It was also agreed upon that the coders would use language and terminology derived from a social cognitive framework so as to maintain interrater consistency.

In the next phase, we used an open coding process in which codes were not created a priori, but rather induced from the narrative data. After transcribing all evaluations into one document, three authors (BH, JP, & SP) coded the evaluations separately. Although coding was guided partially by findings from step 1, codes were developed and modified where needed throughout the coding process. After each author had completed coding of the evaluations, the authors met to discuss coding. Codes were retained only if all three coders agreed on the code, or were modified where appropriate. For example, in instances where one coder applied a code to a piece of text that differed from the code used by the other two coders, all three coders engaged in discussion to determine which code was most appropriate in the context of social cognition. For example, insight, perception or self-attributions might be initially selected as codes, and at the subsequent step of discussing relationships across the codes, the theme of developing of self-acceptance through emotional reflection emerged. After the initial coding process, the emergent codes were further refined using the RQDA package [38] in R [39]; in which prior codes were retained, and additional detailed qualifiers added.

In the third step, data were displayed in a table to facilitate conclusion drawing. Codes were represented on the table rows and participant responses (text) were represented in the column. The three coders met to discuss which codes should be grouped together based on similarity in meaning and the extant literature in the ED field. This process was undergone by first selecting a code word, followed by discussion about which other codes were potentially related. For example, one of the most commonly used codes was “attributions.” Assuming that the frequency of the code word was somewhat related to its importance in indicating a potential theme, additional code words related to “attributions” (e.g., “cognition”) were identified and grouped together (Table 1). Once all code words were grouped based upon their substantive meaning, the three coders searched for themes among the data. As a starting point, themes were considered within the context of the social psychology of cognition and the cognition of social psychology [40].

**Table 1:** List of themes and associated codes

Developing self-acceptance with emotional reflection	Changing expectations with neurosocial knowledge	Bonding and vulnerability in social interaction
Comparison	Assumptions	Acceptance
Expectations	Attitude	Bonding
Identity	Attributions	Burden
Insight	Brain response	Comfort zone
Mindfulness	Cognition	Communication
Perception	ED talk	Help-seeking
Perfectionism	Perspective	In-group
Self-blame	Psychoeducation	Interaction
Self-compassion		Motivation
		Observation Others
		Social support
		Team work Triggering

Finally, after identification of themes, data pertinent to a particular theme were gathered and reviewed. Themes were reviewed both in the context of a single question on the evaluation form and collectively across all questions. Particular attention was given to themes that may be too broad (i.e., comprised of multiple sub-themes) or were not well supported by the data. In the latter case, consideration was made as to whether any themes would best be combined.

The coding and theme extraction process described above was partially informed by the questions presented to participants on the feedback form. For example, one question, “Have you noticed any changes in your general attitudes or attributions since starting the intervention?” was expected to produce participant narrative that aligned with “attitude” and “attribution” codes and thus would allude to a neurosocial knowledge theme. In this way, coders did have an a priori expectation about what themes would emerge. However, the coding and theme extraction process was not guided by a priori codes. While the feedback form was targeted in the questions asked of participants, we remained open to codes and emergent themes that did not align with a priori expectations. Coders met on multiple occasions to discuss the coding process as a way to check biases in our coding and to maintain integrity in the participants’ responses so as to ensure that participant narrative was not unduly coded based on a priori expectations.

## Results

### Participants

Twenty-four participants enrolled and attended at least one group therapy session; the participants were split in four separate cohorts. The participants had AN (n = 11), BN (n = 11), and BED (n = 2), and their age ranged from 19 to 41 years (M = 28.5, SD = 5.76). Most participants identified their race as White (n = 22) and ethnicity as non-Hispanic (n = 20). Four participants identified as Hispanic, one as Black, and one as other. Twenty-one participants provided feedback using the short questionnaire after the last session; twenty completed the longer follow-up questionnaire at 1–4 weeks.

### Overview of results

The thematic analysis of responses to the post-intervention questionnaire and first follow-up resulted in 149 pieces of text, each representing a participant’s complete response to one evaluation item, and 31 codes. In general, multiple codes were attached to a single piece of text. Consistent patterns among the coded data emerged and were contextualized as themes present in ED recovery. After reviewing and grouping codes, three main themes were identified: (1) Developing self-acceptance through emotional reflection, (2) Changing expectations with neurosocial knowledge, and (3) Bonding and vulnerability in social interactions (Table 1; Fig. 1). These data suggest that the intervention targets of self-blame and perspective-taking in relation to social behavior were present in the participants’ evaluations after the intervention and were generally well-received.

### Developing self-acceptance through emotional reflection

After completing the SBPI, many participants described shifts in their self-perception that allowed them to better understand how others might, in turn, perceive them and how they thought about themselves in relation to that. To the participants, this shift in perception allowed them to re-evaluate how they respond to the way others communicate with them and the actions of others. In this respect, participants described having the ability to refrain from over-thinking about others.

How I interpret things (people’s actions) might not be correct. Since this is so, it’s important not to try to read too much into things. Also when people compliment it, they mean it in a positive way and nothing more or less.

Other participants described the important role of self-reflection and the ability to think and react to their own feelings. One participant listed the “primary lesson” from the intervention as, “*Redirecting the negative self-talk. Catching the constant critical thinking towards myself*.” In turn, the ability to self-reflect provided participants with a better sense of how to communicate with themselves and others. This provided a degree of emotional freedom, resulting from their shift in perception. Another participant reflected*, “I’m not excellent at changing or re-directing my negative self-talk; however, I am so much more aware of what is happening in my mind. There is more of a “pause” between think and do/react.”* Many participants commented on their tendency to self-blame and the shift in this thinking after completing the intervention: *“Noticing I am human and there are flaws and challenges I will walk through. Comparing myself to others is wasted time and energy. It is not helpful.”*

Importantly, shifts in self-perception are often understood within the context of self-categorization, in which social processes are explained by the shift from a first-person perspective to a third-person perspective [41, 42]. Our participants described how their shifts in perception increased their feelings of empathy, helping them to understand that others may act for reasons unrelated to themselves or for reasons beyond their control. In turn, the participants’ ability to understand that others’ actions may be unrelated to themselves, allowed them to feel that they are not necessarily the reason for negative actions of those around them*.* One participant reflected, *“Learning about perspective has really stuck with me. I’ve been thinking more about why people may act or do certain things beyond reasons I can see or assume. I’ve been trying to put myself in others’ shoes as well as tell myself not everything people do that is negative is my fault or because of me.”* As a result of the understanding that others sometimes act for reasons beyond their control, many participants described a decreased sense of self-blame and a higher sense of self-compassion and self-acceptance. This idea was articulated by one participant:

The fact that not everything is my fault and I don’t have to take responsibility for someone else’s opinion because they have their own situation.

### Changing expectations with neurosocial knowledge

During the intervention, participants were engaged in a series of psychoeducation segments that provided information about social cognition in general as well as neuropsychological antecedents and consequences to their ED. Participants described developing an increased understanding of the pathology of their ED and how their brain functions in relation to those of healthy controls. With this, participants felt they had a better understanding of the way in which the ED brain operates.

I think the psychoeducational research was very insightful, as well as informative. I enjoyed learning about the depth of my disease and how it can affect my brain's functioning.

Participants enjoyed reflecting on their ED within a more neuropsychological context as well. Some women described this as being coupled with a sense of relief, understanding that their disease is not something to be at fault for and can be managed. As one participant stated:

I like knowing there is science to back up the way I am, it’s not just something in my head. It gives me hope to know if I just keep practicing the things I learned that I might be able to not feel this way forever or be able to control it better.

Some participants found the psychoeducation and the homework to be the most helpful parts of the intervention, in particular the social perspective-taking exercises.

The psychoeducation allowed me to realize the importance of thinking about things with an outside perspective. The homework put the psychoeducation to use and applied it on a personal level which allowed for more growth.

The psychoeducation components focused on creating a better understanding of both attribution biases and perspective-taking; attribution bias is important in self-regulation and impacts on social expectancies [40]. Many participants described a tendency to make assumptions that others’ actions were caused because of something they had done wrong. In learning about cognitive biases, participants felt they were better able to understand that others’ actions are often unrelated to themselves. For example, one participant expressed:

Being aware of/processing different people’s reactions or perceptions of a situation. The fact that not everything is my fault and I don’t have to take responsibility for someone else’s opinion because they have their own situation.

Likewise another participant reflected*:*

I have been allowing to give myself the benefit of the doubt more often when someone acts negatively towards me like it isn’t always something I’ve done wrong or my fault.

The relationship between one’s mental representations of the world around them and other people acting in their environment, was further explored in the psychoeducation with the participants, with an emphasis on how different people have different perspectives. This understanding of attribution biases and perspective-taking led many women to reflect on how their attribution biases might impact their relationships and/or interaction with others*.* This was further contextualized by participants who suggested that the uniqueness of certain situations they encounter should result in different reactions, both from themselves and the other involved persons. A participant reflected, *“I feel like I understand assumptions more, about how you can’t always change them and may just have to accept that people think or feel a certain way, but you don’t have to agree. I think more about my own reactions and judgments as well.”*

### Group bonding and vulnerability in social interaction

The importance of bonding with others and being vulnerable in social interactions was a key takeaway from the intervention. Participants remarked on the benefits of bonding with women who also have EDs, and describing communication improvements from the activities. In so doing, participants felt they were better prepared to ask for help and find support in others who share similar journeys. Accordingly, one participant wrote*, “I really enjoyed doing team building activities with the girls. What I took away from that experience is that we need each other's support, in one form or another. Just being around girls that I know struggle with similar issues made me more motivated for recovery.”*

Similarly, teamwork and enjoying cooperative tasks were highlighted repeated by participants: “*The art and teamwork aspect were the most beneficial*”. One participant noted that the group changed how she approached social interactions, “[Most valuable?] *The art projects. It was a new process for me and made me think about working with others in a different way*.”

Although the bonding that occurred made some participants feel vulnerable, they felt that this vulnerability was useful in building trust with one another. Participants described a decreased sense of “*feeling in competition*” with one another, and instead, bonding over commonalities. This was particularly highlighted in the art tasks which they felt explored complex tendencies and characteristics shared among the women. As one described:

This also engaged us a lot beyond being vulnerable and talking mostly about ED topics. There were points of vulnerability, but it also felt bonding to do silly tasks that took teamwork and trust.

Even some who did not enjoy the art therapy, noted utility from learning from the group interactions: *“Art therapy was not my favorite, however it was effective in me addressing social dynamics with my therapist, so maybe it was also helpful.*” The team building aspect of the intervention led participants to feel encouraged to “*step out of their comfort zone*” and work with others.

### Exclusion of ED symptom discussion

Participants were specifically asked about whether the rule to avoid personal ED symptom/behavior discussion was helpful or not and why in the feedback questionnaire. While the responses aligned with themes described previously, we discuss separately to enrich our understanding of how patients with EDs perceive their experiences in groups with and without ED talk.

In response to this query, many participants detailed prior experiences with negative group interactions around ED behaviors, and appreciated the guideline that personal ED discussion would be avoided as these were deemed triggering. One participant said “*Most times sitting around and talking about ED behaviors actually makes me want to practice my eating disorder*” and another said “*[Discussing ED behaviors] can turn into triggering, unhelpful war stories*”. A third stated: *“If it were allowed, I feel like people would feed off of each other’s eating behaviors and it wouldn’t feel as if we were productively changing behavior.”* Another reported: “*So helpful! Because I have noticed through treatment that people with EDs can get caught up in basically segregating with others who are more “like them”. But this intervention kept everyone together.*” Another noted “*I think it was helpful because I’m sure we all have different experiences and it would be hard if one person took control of the conversation to fit their needs*”. One who found it unhelpful noted wanting to learn about the other participants’ illnesses as a means to bonding “*I also was hoping to hear what other girls in the group were struggling with in their eating disorders*” and another “*I think it would be good to hear some people are not alone in this and as a group we could help each other out*”. All of these comments reflect on the “Bonding and vulnerability in social interactions” theme previously detailed, but adds the nuance that many of these participants had experienced negative social interactions related to ED symptom discussions in other settings.

Some interest in discussing ED behaviors was related to a desire for more specific information about ED pathology. “*Not talking about it makes it more general and you can apply what you learned to all things, not just eating. But if we had discussed how it related to eating disorder I might feel more empowered in that one specific area*”. And another “*This gave us the opportunity to get educated on life, environmental, and society, and how it effects our minds and EDs*.” Another person enjoyed the opportunity to focus on other things: “*I liked that part of it. Once you get on that topic [personal ED symptoms], it can take up the whole session and still not be resolved or finished. It was really good to talk about the brain, the art projects, and why things are, and offer and hear the others’ feedback.*” These types of comments reflected the desire to learn more about psychology and brain function, aligning with the “Changing expectations with neurosocial knowledge” theme.

A few comments suggested that avoiding eating discussion was helpful by diminishing the importance of the ED identity, fitting the first theme of “Developing self-acceptance through emotional reflection”. For example, one said “*Helpful – because it put the focus more on underlying causes rather than the eating disorder. It eliminated some of the feelings of guilt and helped me be more open*.” And another stated that “*It felt like we had more to ourselves than just the ED*” because ED talk was not involved in developing the relationships with others in the group, allowing the person’s identity to expand beyond the ED.

However, one participant disagreed, stating that “*I think it was unhelpful because ED behavior – frequency, intensity, duration – is the main meter of recovery in action. And you can’t know how you’re doing if you don’t have good data/troubleshoot trends in data*”. And a few suggested benefits from avoiding ED discussion in the group depend on specific stage of recovery: “*I think it depends on the participants. Some were wanting to talk about it because they wanted to see how other people go through it*.” One participant believed it was “*Unhelpful because I feel like I didn’t get the opportunity to really discuss what I felt are the core issues to my eating disorder.”* And another reported that “*It [not talking about ED] was helpful for me because my main issues right now are not my eating disorder, but rather depression and anxiety.*”

## Discussion

The Self-Blame and Perspective-Taking intervention (SBPI) was designed to include explicit discussion of brain-based social perceptual challenges observed in EDs in concert with a group that experientially targeted those social challenges and simultaneously provided a positive social experience. The themes identified in the qualitative analyses suggest the neurosocial targets were effectively taught to participants. Further, this knowledge was associated with reported improvements in the emotional state and self-acceptance of participants. In addition, the participants described feeling that the SBPI was a positive social experience. In sum, both the neurosocial information and the interactions within the group led to a perceived positive impact in the participants’ lives. These descriptions add depth to our understanding of the quantitative results which support more positive self-concept and reduced clinical symptoms following the intervention. Psychosocial functioning is a key component in ED recovery [43]. In a literature review assessing themes observed in lived experiences with personal recoveries from EDs, Wetzler and colleagues identified six components: supportive relationships, hope, identity, meaning and purpose, empowerment, and self-compassion [44]. The feedback from the SBPI revealed three main themes: (1) Developing self-acceptance through emotional reflection, (2) Changing expectations with neurosocial knowledge, and (3) Bonding and vulnerability in social interactions; all themes are closely related to the supportive relationships, identity, empowerment, and self-compassion described in that synthesis of personal recovery experiences.

The cognitive-affective interpersonal maintenance model for EDs presupposes that social-emotional dysregulation is coupled with cognitive distortions and interpersonal difficulties [45]. The themes identified here are highly consistent with the key features of the maintenance model for EDs. We have nested our themes in Fig. 1, placing Self-Acceptance at the core, as the basic question about how an individual feels about themselves is a concept not always accessible verbally or obvious to external viewers. Neurosocial knowledge reflects the abstract information about social function; differences in our abstract knowledge can change both one’s interactions with others as well as one’s internal emotional beliefs about oneself. Finally, the outer rim of Social Interactions relates to the actual experiences working with other group members. Thinking of this more broadly, the sum of social interactions over a lifetime impacts both our beliefs about social dynamics as well as self-acceptance for all people.

The SBPI did not permit discussion of ED behaviors. This element received mixed qualitative feedback, but our rationale for this guideline developed from our pre-study clinical conversations with patients. First, potential participants described withdrawing from ED support groups because discussion of ED cognitions and behaviors could be triggering. The guideline of no ED discussions was set to encourage attendance for patient-participants that previously avoided groups. Second, the goal of the SBPI was to create a supportive social environment conducive to team-building. Symptom discussion can engender competition and comparison, which can interfere with social support [7, 46]. The data provided here, support a need for clinicians to recognize that discussion of ED behaviors in groups can be triggering and evaluate its need in other settings. Another qualitative study examining motivation for recovery found that non-judgmental healthcare providers who could see beyond weight and eating to the emotional well-being of their patients were most helpful [47]. The SBPI provided an opportunity for patients to work with peers and clinicians without focusing overtly on the ED behaviors.

Several limitations warrant discussion. First, only participants interested in a group intervention participated, likely biasing our analysis towards positive feedback. We were not able to obtain feedback from participants who declined to participate or withdrew. Those responses might provide different perspectives on intervention feasibility or illuminate areas for improvement. Our sample size was adequate for qualitative investigation but the perspectives of participants may not represent the full range of possible experiences with the intervention, as feedback was collected with a semi-structured questionnaire. This supported our aim to assess feasibility about the targets as well as acceptability of the experience, but likely limited the variety and depth of participant responses. Additionally, we did not conduct member checking (i.e., ask the participants if the themes we found were accurate). In an ideal qualitative study, we would have used respondent validation to verify that our results reflected the true thoughts and emotions of our participants. Our queries were designed to promote responses related to the targets of the intervention and its components, potentially restricting the information offered. Finally, our study sample only included females, and results may not generalize to males or nonbinary individuals with EDs.

## Conclusions

In summary, participants in the SBPI endorsed learning and incorporating both intervention targets, self-attributions and perspective-taking, into their lives. The intervention as a whole was also perceived positively both due to benefits from learning about those targets and enjoying the structured social interactions. The SBPI may help meet a clinical need for social connection that can be lacking when patients with EDs transition to outpatient care. Social support was identified as a key element to recovery in a qualitative study reviewing weblogs of recovered individuals [48]. Specifically, in inpatient, residential, and partial hospital programs, patients often develop a strong sense of community from the peer support present in these environments [49–51]. When patients with EDs move to outpatient care, the ability to obtain similar social support is difficult. Many try in-person and online support groups, but these options can trigger ED symptoms [52–54]. Prior research on patient perspectives indicates that addressing interpersonal function and relationships is an important component of ED recovery [5, 6]. The SBPI may fill a treatment gap for outpatients with EDs by both targeting social challenges common in EDs as well as providing social support.

## Data Availability

Data and materials are available on request.

## Abbreviations

AN: Anorexia nervosa
BN: Bulimia nervosa
CREST: Cognitive Remediation and Emotion Skills Training
CBT-E: Enhanced cognitive behavioral therapy
ED: Eating disorder
RO-DBT: Radically Open Dialectal Behavioral Therapy
RQDA: R package for Qualitative Data Analysis
SBPI: Self-Blame Perspective-Taking Intervention

## References

[1] Jenkins J, Ogden J (2011). Becoming 'whole' again: a qualitative study of women's views of recovering from anorexia nervosa. Eur Eat Disord Rev J Eat Disord Assoc..

[2] Winkler LA, Christiansen E, Lichtenstein MB, Hansen NB, Bilenberg N, Stoving RK (2014). Quality of life in eating disorders: a meta-analysis. Psychiatry Res.

[3] Keel PK, Dorer DJ, Franko DL, Jackson SC, Herzog DB (2005). Postremission predictors of relapse in women with eating disorders. Am J Psychiatry.

[4] McFarlane T, Olmsted MP, Trottier K (2008). Timing and prediction of relapse in a transdiagnostic eating disorder sample. Int J Eat Disord.

[5] de Vos JA, LaMarre A, Radstaak M, Bijkerk CA, Bohlmeijer ET, Westerhof GJ (2017). Identifying fundamental criteria for eating disorder recovery: a systematic review and qualitative meta-analysis. J Eat Disord.

[6] Linville D, Brown T, Sturm K, McDougal T (2012). Eating disorders and social support: perspectives of recovered individuals. Eat Disord.

[7] Beresin EV, Gordon C, Herzog DB (1989). The process of recovering from anorexia nervosa. J Am Acad Psychoanal.

[8] Cockell SJ, Zaitsoff SL, Geller J (2004). Maintaining change following eating disorder treatment. Prof Psychol Res Pract.

[9] Pettersen G, Rosenvinge JH (2002). Improvement and recovery from eating disorders: a patient perspective. Eat Disord.

[10] Rorty M, Yager J, Rossotto E (1993). Why and how do women recover from bulimia nervosa? The subjective appraisals of forty women recovered for a year or more. Int J Eat Disord.

[11] Patel K, Tchanturia K, Harrison A (2016). An exploration of social functioning in young people with eating disorders: a qualitative study. PLoS ONE.

[12] Harrison A, Watterson SV, Bennett SD (2018). An experimental investigation into the use of eye-contact in social interactions in women in the acute and recovered stages of anorexia nervosa. Int J Eat Disord.

[13] Cardi V, Mallorqui-Bague N, Albano G, Monteleone AM, Fernandez-Aranda F, Treasure J (2018). Social difficulties as risk and maintaining factors in anorexia nervosa: a mixed-method investigation. Front Psychiatry.

[14] McAdams CJ, Harper JA, Van Enkevort E (2018). Mentalization and the left inferior frontal gyrus and insula. Eur Eat Disord Rev J Eat Disord Assoc.

[15] Morrison T, Waller G, Lawson R (2006). Attributional style in the eating disorders. J Nerv Ment Dis.

[16] Wittorf A, Giel KE, Hautzinger M, Rapp A, Schonenberg M, Wolkenstein L (2012). Specificity of jumping to conclusions and attributional biases: a comparison between patients with schizophrenia, depression, and anorexia nervosa. Cogn Neuropsychiatry.

[17] Bora E, Kose S (2016). Meta-analysis of theory of mind in anorexia nervosa and bulimia nervosa: a specific Impairment of cognitive perspective taking in anorexia nervosa?. Int J Eat Disord.

[18] McAdams CJ, Krawczyk DC (2013). Neural responses during social and self-knowledge tasks in bulimia nervosa. Front Psychiatry.

[19] McAdams CJ, Krawczyk DC (2014). Who am I? How do I look? Neural differences in self-identity in anorexia nervosa. Soc Cogn Affect Neurosci.

[20] McAdams CJ, Lohrenz T, Montague PR (2015). Neural responses to kindness and malevolence differ in illness and recovery in women with anorexia nervosa. Hum Brain Mapp.

[21] Luo Y, Mendoza C, Pelfrey S, Lohrenz T, Gu X, Montague PR, McAdams CJ (2021). Elevated Neurobehavioral Responses to Negative Social Interactions in Women With Bulimia Nervosa. Biol Psychiatry Cogn Neurosci Neuroimaging..

[22] Cardi V, Tchanturia K, Treasure J (2018). Premorbid and illness-related social difficulties in eating disorders: an overview of the literature and treatment developments. Curr Neuropharmacol.

[23] Tchanturia K, Doris E, Fleming C (2014). Effectiveness of Cognitive Remediation and Emotion Skills Training (CREST) for anorexia nervosa in group format: a naturalistic pilot study. Eur Eat Disord Rev J Eat Disord Assoc.

[24] Tchanturia K, Doris E, Mountford V, Fleming C (2015). Cognitive Remediation and Emotion Skills Training (CREST) for anorexia nervosa in individual format: self-reported outcomes. BMC Psychiatry.

[25] Lynch TR, Gray KL, Hempel RJ, Titley M, Chen EY, O'Mahen HA (2013). Radically open-dialectical behavior therapy for adult anorexia nervosa: feasibility and outcomes from an inpatient program. BMC Psychiatry.

[26] Chen EY, Segal K, Weissman J, Zeffiro TA, Gallop R, Linehan MM (2015). Adapting dialectical behavior therapy for outpatient adult anorexia nervosa—a pilot study. Int J Eat Disord.

[27] Miniati M, Callari A, Maglio A, Calugi S (2018). Interpersonal psychotherapy for eating disorders: current perspectives. Psychol Res Behav Manag.

[28] Berends T, Boonstra N, van Elburg A (2018). Relapse in anorexia nervosa: a systematic review and meta-analysis. Curr Opin Psychiatry.

[29] Brodrick B, Harper JA, Van Enkevort E, McAdams CJ (2019). Treatment utilization and medical problems in a community sample of adult women with anorexia nervosa. Front Psychol.

[30] Levinson CA, Brosof LC, Vanzhula IA, Bumberry L, Zerwas S, Bulik CM (2017). Perfectionism group treatment for eating disorders in an inpatient, partial hospitalization, and outpatient setting. Eur Eat Disord Rev J Eat Disord Assoc.

[31] Mac Neil BA, Hudson CC (2018). Patient experience and satisfaction with acceptance and commitment therapy delivered in a complimentary open group format for adults with eating disorders. J Patient Exp.

[32] Hunt BJ, Hagan WS, Pelfrey S, Mericle S, Harper JA, Palka JM (2021). Pilot data from the Self-Blame and Perspective-Taking Intervention for eating disorders. J Behav Cogn Ther.

[33] Sheehan DV, Lecrubier Y, Sheehan KH, Amorim P, Janavs J, Weiller E (1998). The Mini-International Neuropsychiatric Interview (M.I.N.I.): the development and validation of a structured diagnostic psychiatric interview for DSM-IV and ICD-10. J Clin Psychiatry.

[34] Sysko R, Glasofer DR, Hildebrandt T, Klimek P, Mitchell JE, Berg KC (2015). The eating disorder assessment for DSM-5 (EDA-5): development and validation of a structured interview for feeding and eating disorders. Int J Eat Disord.

[35] Wechsler D (2011). Wechsler Abbreviated Scale of Intelligence-Second Edition (WASI-II).

[36] Hunt B, Hagan WS, Pelfrey S, Mericle S, Harper JA, Palka JM (2021). Pilot data from the Self-Blame and Perspective-Taking Intervention for eating disorders. J Behav Cogn Ther.

[37] Braun V, Clarke V (2006). Using thematic analysis in psychology. Qual Res Psychol.

[38] Huang R. RQDA: R-based qualitative data analysis. R package version 0.2-8. 2016. http://rqda.r-forge.r-project.org/.

[39] Team RC (2020). R: a language and environment for statistical computing.

[40] Higgins ET (2000). Social cognition: learning about what matters in the social world. Eur J Soc Psychol.

[41] Kim Y-H, Chiu C-Y, Cho S, Au EWM, Kwak SN (2014). Aligning inside and outside perspectives of the self: a cross-cultural difference in self-perception. Asian J Soc Psychol.

[42] Turner JC, Oakes PJ, Haslam SA, McGarty C (1994). Self and collective: cognition and social context. Pers Soc Psychol Bull.

[43] Bardone-Cone AM, Harney MB, Maldonado CR, Lawson MA, Robinson DP, Smith R (2010). Defining recovery from an eating disorder: conceptualization, validation, and examination of psychosocial functioning and psychiatric comorbidity. Behav Res Ther.

[44] Wetzler S, Hackmann C, Peryer G, Clayman K, Friedman D, Saffran K (2020). A framework to conceptualize personal recovery from eating disorders: a systematic review and qualitative meta-synthesis of perspectives from individuals with lived experience. Int J Eat Disord.

[45] Treasure J, Schmidt U (2013). The cognitive-interpersonal maintenance model of anorexia nervosa revisited: a summary of the evidence for cognitive, socio-emotional and interpersonal predisposing and perpetuating factors. J Eat Disord.

[46] Warin MJ (2006). Reconfiguring relatedness in anorexia. Anthropol Med.

[47] Venturo-Conerly KE, Wasil AR, Dreier MJ, Lipson SM, Shingleton RM, Weisz JR (2020). Why I recovered: a qualitative investigation of factors promoting motivation for eating disorder recovery. Int J Eat Disord.

[48] Smethurst L, Kuss D (2018). 'Learning to live your life again': an interpretative phenomenological analysis of weblogs documenting the inside experience of recovering from anorexia nervosa. J Health Psychol.

[49] de la Rie S, Noordenbos G, Donker M, van Furth E (2006). Evaluating the treatment of eating disorders from the patient's perspective. Int J Eat Disord.

[50] Eli K (2014). Between difference and belonging: configuring self and others in inpatient treatment for eating disorders. PLoS ONE.

[51] Smith K, Lesser J, Brandenburg B, Lesser A, Cici J, Juenneman R (2016). Outcomes of an inpatient refeeding protocol in youth with anorexia nervosa and atypical anorexia nervosa at Children's Hospitals and Clinics of Minnesota. J Eat Disord.

[52] Koski JP (2014). 'I'm just a walking eating disorder': the mobilisation and construction of a collective illness identity in eating disorder support groups. Sociol Health Illn.

[53] Ransom DC, La Guardia JG, Woody EZ, Boyd JL (2010). Interpersonal interactions on online forums addressing eating concerns. Int J Eat Disord.

[54] Vandereycken W (2011). Can eating disorders become 'contagious' in group therapy and specialized inpatient care?. Eur Eat Disord Rev J Eat Disord Assoc.

